# A Multiscale Model of Oxidation Kinetics for Cu-Based Oxygen Carrier in Chemical Looping with Oxygen Uncoupling

**DOI:** 10.3390/ma12071170

**Published:** 2019-04-10

**Authors:** Hui Wang, Zhenshan Li, Ningsheng Cai

**Affiliations:** 1 Key Laboratory for Thermal Science and Power Engineering of Ministry of Education, Department of Energy and Power Engineering, Tsinghua University, Beijing 100084, China; thu_wh@126.com (H.W.); cains@tsinghua.edu.cn (N.C.); 2 Tsinghua University-University of Waterloo Joint Research Center for Micro/Nano Energy & Environment Technology, Tsinghua University, Beijing 100084, China

**Keywords:** oxygen carrier, multiscale model, product island, oxidation kinetics

## Abstract

Copper oxide is one of the promising oxygen carrier materials in chemical looping with oxygen uncoupling (CLOU) technology, cycling between Cu_2_O and CuO. In this study, a multiscale model was developed to describe the oxidation kinetics of the Cu-based oxygen carrier particle with oxygen, including surface, grain, and particle scale. It was considered that the solid product grows with the morphology of disperse islands on the grain surface, and O_2_ contacts with two different kinds of grain surfaces in the grain scale model, that is, Cu_2_O surface (solid reactant surface) and CuO surface (solid product surface). The two-stage behavior of the oxidation reaction of the Cu-based oxygen carrier was predicted successfully using the developed model, and the model results showed good agreement with experimental data in the literature. The effects of oxygen partial pressure, temperature, and particle structure on the oxidation performance were analyzed. The modeling results indicated that the transition of the conversion curve occurs when product islands cover most part of the grain surface. The oxygen partial pressure and particle structure have an obvious influence on the duration time of the fast reaction stage. Furthermore, the influence of the external mass transfer and the change of effectiveness factor during the oxidation reaction process were discussed to investigate the controlling step of the reaction. It was concluded that the external mass transfer step hardly affects the reaction performance under the particle sizes normally used in CLOU. The value of the effectiveness factor increases as the reaction goes by, which means the chemical reaction resistance at grain scale increases resulting from the growing number of product islands on the grain surface.

## 1. Introduction

Chemical looping combustion (CLC) is a new combustion technology [1,2], where oxygen carriers are used to transport oxygen from the air reactor to the fuel reactor through the redox cycle. Compared with traditional CO_2_ capture technologies, such as pre-combustion capture [3], post-combustion capture [4], and oxy-fuel combustion [5], CLC technology has obvious advantages in reducing the energy consumption of CO_2_ capture. The chemical looping with oxygen uncoupling (CLOU) concept [6] is based on CLC technology, where the oxygen carriers have oxygen release capacity. Solid fuel can react directly with oxygen released from oxygen carriers in the fuel reactor to improve combustion efficiency. The research results of Mattisson et al. [6] show that when petroleum coke is used as fuel, the conversion of the CLOU process is 50 times higher than that of traditional CLC process. Subsequently, many researchers further explored the oxygen carriers suitable for CLOU technology [7,8,9,10,11,12,13,14,15,16].

The Cu-based oxygen carrier was reported to have a strong oxygen release capacity and fast reaction rate in other research [9,10,11,12,13,14,15,16], and the corresponding redox pair was CuO/Cu_2_O. Both the oxidation and reduction of Cu-based oxygen carriers are gas-solid reactions. There are a large number of studies on the oxidation or reduction kinetics of Cu-based oxygen carriers. de Diego [11], Goldstein [12], and Gayn [13] used pure CuO as oxygen carriers to conduct the kinetic tests. In addition, researchers [14,15,16] used inert carrier materials and preparation methods to prepare Cu-based oxygen carriers with improved cyclic stability. It was widely found in the experimental results that there is a transition of the kinetics from the initial fast stage to the second slower stage in the conversion curve of the Cu_2_O oxidation reaction [11,13,14,15,16].

To explain the kinetic behavior of the gas-solid reaction kinetics of the oxygen carrier, many kinds of models were developed. In the research of Clayton et al. [16], two apparent models, pore-blocking model and Avrami-Erofeev model, were used for the oxidation reaction of Cu_2_O in the lower temperature range (below 700 °C) and higher temperature range (above 800 °C), respectively. García-Labiano et al. [17] and Maya et al. [18] used the grain model to predict the reaction behavior of oxygen carriers. The grain model assumes the particle to be a spherical porous solid particle that consists of numerous small grains within, and each of these grains is described using the unreacted shrinking core model. In addition, Dennis et al. [19] and Liu et al. [20] utilized the pore model to explain the kinetic performance of gas-solid reaction. Nevertheless, the models mentioned above were all focused on the particle scale step, including the external and internal mass transfer, while these models did not consider the microscopic reaction steps taking place on the grain surface and the growth of the solid product, which play an important role in the gas-solid reaction process [21]. Therefore, these models cannot explain the transition phenomenon from the initial fast stage to the second slower stage in the conversion curve from the view of the microscopic reaction process.

In the review paper of Gattinoni et al. [22], recent surface science, spectroscopy, and atomic computation work performed to understand the copper oxidation from the microscopic point of view was summarized and discussed. A good amount of computational work has been performed on the formation of copper oxides, providing important information on surface reaction process. However, few experimental studies are available to either confirm or disprove some computational results obtained at the atomic scale, and the nucleation details of the oxide islands are still unknown. Also, Zhang et al. [23] and Yu et al. [24] applied density functional theory to investigate the oxygen adsorption and dissociation process on the Cu_2_O surfaces. The calculated results showed that the presence of oxygen vacancy on the surface exhibited a strong chemical reactivity towards the dissociation of O_2_. Recently, it was found that the solid product showed dispersed and three-dimensional morphology on the solid reactant surface [25,26,27,28]. In addition, a rate equation theory for Fe oxidation was developed to describe the nucleation and growth process of the solid product [25]. However, the above research at microscopic scale did not consider the steps involved at the particle scale, such as particle structure change, external mass transfer, and internal mass transfer, thus could not explain the phenomenon at the macroscopic scale and the controlling mechanism of the reaction.

The gas-solid reaction of the Cu-based oxygen carrier with oxygen is a multiscale behavior. It is necessary to study the reaction process from multiscale points of view. In this study, a multiscale model, including surface, grain, and particle scale, was established to describe the oxidation reaction of Cu-based oxygen carrier, and the developed model was validated with experimental data in the literature. Then, the model was used to analyze the effects of oxygen partial pressure, temperature, and particle structure on the oxidation reaction behaviors. Further, the controlling step of the oxidation reaction of the Cu-based oxygen carrier particle was discussed.

## 2. Mathematical Model

In this study, a multiscale model was developed to describe the oxidation reaction of the Cu-based oxygen carrier. The oxidation reaction of the Cu-based oxygen carrier is

(1) $${\mathrm{Cu}}_{2}O+\frac{1}{2}O_{2}\to 2\mathrm{CuO}$$

**Figure 1:**
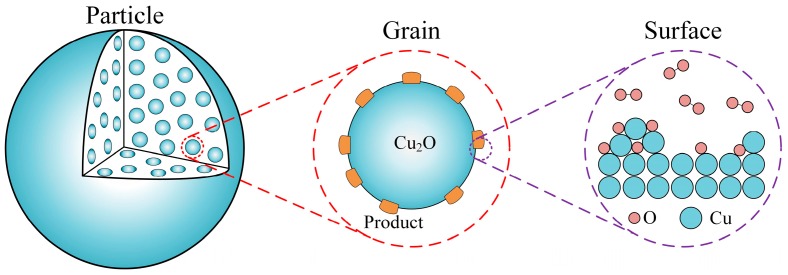
Schematic diagram of the multiscale model.

As shown in Figure 1, the oxidation process of the oxygen carrier particle was described at multiscale, that is, surface scale, grain scale, and particle scale. The oxygen carrier particle was considered as spherical porous media, which is composed of a matrix of spherical nonporous grains, and O_2_ could diffuse into the particle. The interaction of O_2_ with Cu_2_O occurs on the grain surface, and the solid product will grow on the grain surface, which can cover the grain surface and result in the change of grain size and an increase of gas diffusion resistance. In the thermogravimetric analysis (TGA) experiments of Clayton et al. [16], it was pointed out that the Cu-based oxygen carrier particle could be considered isothermal during the oxidation process. In addition, in the study of García-Labiano et al. [17], the coupled energy equations were considered in their grain model, and it was also concluded that the oxygen carrier particle could be considered isothermal for most of the reactions in a chemical looping combustion process. Therefore, based on the above studies, the temperature inside the oxygen carrier particle is considered isothermal in the model of this work.

### 2.1. Model at Surface Scale

Up to now, many theoretical and experimental studies have been reported to investigate the O_2_ adsorption on Cu-based metal oxide surface [22,23,24,29,30,31,32,33,34]. When the Cu_2_O surface is exposed to oxygen, oxygen molecules would be absorbed on the surface and generate adsorbed oxygen and oxygen ions [29,30,31,32,33,34]. On the Cu_2_O-O_2_ interface, the surface reaction involves several steps [31,32,33]: (R1) O_2_ gas molecules are adsorbed on the metal oxide surface and decomposed into adsorbed oxygen, O(ads); (R2) O(ads) takes electrons from the metal cations (Cu^+^) and forms chemically adsorbed oxygen, O^−^(chem); (R3) O^−^(chem) further reacts with electrons to form lattice oxygen, O^2−^(latt). During the reaction, the oxygen from the most outward lattice position would diffuse in the crystal structure to replenish oxygen vacancies. This reaction process can be described as follows.

(R1) $$O_{2}(g)+2*\overset{k_{1}}{\underset{k_{-1}}{⇄}}2O(\mathrm{ads})$$

(R2) $$2O(\mathrm{ads})+2e^{-}\overset{k_{2}}{\underset{k_{-2}}{⇄}}2O^{-}\left(\mathrm{chem}\right)$$

(R3) $$2O^{-}\left(\mathrm{chem}\right)+2e^{-}\overset{k_{3}}{\to }2O^{2-}\left(\mathrm{latt}\right)$$

The reaction rate of step (R1)–(R3) can be given by

(2) $$r_{R1}=k_{1}\left(P_{O_{2}}-P_{e}\right){\theta_{s}}^{2}-k_{-1}{\theta_{O(\mathrm{ads})}}^{2}$$

(3) $$r_{R2}=k_{2}{\theta_{O(\mathrm{ads})}}^{2}-k_{-2}{\theta_{O(\mathrm{chem})}}^{2}$$

(4) $$r_{R3}=k_{3}{\theta_{O(\mathrm{chem})}}^{2}$$

where $k_{i},\;i=\pm 1,\pm 2,3$ is the reaction rate constant, and the surface site densities are lumped in the reaction rate constant. $\theta_{s},\theta_{O(\mathrm{ads})},\theta_{O(\mathrm{chem})}$ are the coverage ratios of active sites, O(ads), O^−^(chem) on the surface, respectively, which satisfy

(5) $$\theta_{s}+\theta_{O(\mathrm{ads})}+\theta_{O(\mathrm{chem})}=1$$

$P_{e}$ is the equilibrium partial pressure of O_2_, which is expressed as [16]

(6) $$P_{e}=6.057\cdot 10^{-11}\exp\left[0.02146\left(T-273\right)\right]$$

Assuming that R1 and R2 are in chemical equilibrium, $r_{R1}$ and $r_{R2}$ are much less than $r_{R3}$, and hardly zero. Therefore, the reaction rate of R3 could be obtained by combining Equations (2)–(5):

(7) $$r_{R3}=\frac{k_{3}\frac{k_{1}}{k_{-1}}\frac{k_{2}}{k_{-2}}\left(P_{O_{2}}-P_{e}\right)}{{\left[1+\sqrt{\frac{k_{1}}{k_{-1}}\left(P_{O_{2}}-P_{e}\right)}+\sqrt{\frac{k_{1}}{k_{-1}}\frac{k_{2}}{k_{-2}}\left(P_{O_{2}}-P_{e}\right)}\right]}^{2}}$$

In the case of $\frac{k_{1}}{k_{-1}}\left(P_{O_{2}}-P_{e}\right)<<1$, there is

(8) $$r_{R3}=k\left(C-C_{e}\right),\;k=k_{3}\frac{k_{1}}{k_{-1}}\frac{k_{2}}{k_{-2}}R_{g}T$$

where C is the oxygen gas concentration, $C_{e}$ is the equilibrium oxygen concentration, $R_{g}$ is the gas constant, and T is the temperature.

The reaction rate constants involved in the expression of $r_{R3}$ can be calculated through the atomic computation, as discussed in the review paper of Gattinoni et al. [22]. The surface scale model described above could provide the link between microscopic surface reaction and macroscopic kinetics. However, the atomic computation on the reaction rate constants is not the focus of this work. The value of *k* is considered as an adjustable parameter in this work.

### 2.2. Model at Grain Scale

**Figure 2:**
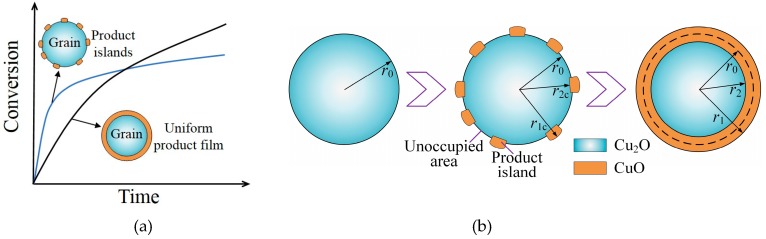
(**a**) Comparison of the product-islands-based model and traditional grain model; (**b**) Schematic diagram of the solid product morphology on grain surface during the oxidation reaction.

In the traditional grain models [17,18], the solid product is assumed to form and grow in a uniform layer-by-layer mode on the grain surface, and the morphology of the solid product is a nonporous film that covers the unreacted core. The theory of a critical product layer thickness [35] is now used in most grain models to explain the end of the fast reaction period. However, because of the simplified description of solid product formation and growth involved in the initial stage, the traditional grain models cannot explain the kinetics well. The typical conversion curve of oxidation of the Cu-based oxygen carrier shows the two-stage shape. The initial stage of the oxidation reaction is fast, and a high conversion of the particle will be achieved in a relatively short time. The fast stage is followed by a slower stage, where the conversion increases slowly. Because of the assumption of uniform solid product film growth, the traditional grain model cannot describe the transition behavior of the reaction kinetics, as shown in Figure 2a.

By using atomic force microscopy (AFM) and a single crystal sample, it has been proposed in several studies that during the gas-solid reaction process, the solid product grows as dispersed, three-dimensional islands rather than a uniform continuous product layer [25,26,27,28]. All islands show similar shape, which can be explained by the Wuff construction theory [36], and the size of islands increases with the oxidation reaction time [25]. Recently, a rate equation theory of metal oxidation was developed to calculate the solid product nucleation and growth rates [25]. It was proposed that the critical size of product islands is temperature-dependent [37]. Therefore, the assumption of a uniform product film on the grain surface was replaced with the product island morphology in the model, as shown in Figure 2a. As the reaction goes on, the product islands will cover the grain surface and hinder the direct contact of the solid reactant with O_2_, as shown in Figure 2b, which slows down the reaction rate significantly and results in the transition of the kinetics from the fast stage to the second slower stage.

It is considered that O_2_ contacts with two different kinds of grain surfaces in the model, that is, Cu_2_O surface (solid reactant surface) and CuO surface (solid product surface) [22]. In the case of O_2_ contacting Cu_2_O surface, the reaction described in Section 2.1 will take place directly on this surface, and product islands will grow on the grain surface meanwhile. The size of the new product island was considered as a critical value of $r_{2c}$ and $r_{1c}$, as shown in Figure 2b, where $r_{2c}$ is the critical grain radius of Cu_2_O/CuO interface and $r_{1c}$ is the critical grain radius of CuO/O_2_ interface. The critical solid reactant layer thickness is described as $h_{c}=r_{0}-r_{2c}$, where $r_{0}$ is the initial grain radius. A key parameter at the grain scale in the model is the ratio of the unoccupied area on the grain surface, which is denoted as δ. By considering the reaction rate of solid reactant and the change rate of the unoccupied area, the expression of δ can be given as [38]

(9) $$\delta =\exp\left[\frac{-2V_{{\mathrm{Cu}}_{2}O}^{M}k\left(C-C_{e}\right)}{h_{c}}t\right]$$

where $V_{{\mathrm{Cu}}_{2}O}^{M}$ is the molar volume of Cu_2_O (23.87 cm^3^/mol), $h_{c}$ is the critical solid reactant layer thickness, and t is the reaction time.

In the case of O_2_ contacting solid product, the charged particles diffusion through the product layer will occur, which can be explained by the Wagner theory [39]. According to the Wagner theory, the diffusion flow rate of metal cations moving through the product layer should be equal to the diffusion flow rate of anions and electrons moving through the product layer to ensure charge conservation. The reaction of metal cations (Cu^+^) and oxygen happens on the Cu_2_O/CuO interface. As the reaction goes on, the size of the solid product will increase and be larger than the critical size, as shown in Figure 2b, where $r_{2}$ is the grain radius of Cu_2_O/CuO interface and $r_{1}$ is the grain radius of CuO/O_2_ interface. In addition, the number of product islands will increase and finally product islands will cover the grain surface completely. By integrating the diffusion flow rate of metal cations (Cu^+^) through the product layer with the change rate of grain size, the change rate of grain size can be obtained as [38]

(10) $$\frac{dr_{2}}{dt}=-\frac{2V_{{\mathrm{Cu}}_{2}O}^{M}\frac{{r_{1}}^{2}}{{r_{2}}^{2}}k\left(C-C_{e}\right)}{r_{1}\left(\frac{r_{1}}{r_{2}}-1\right)\frac{k\left(C-C_{e}\right)}{D_{s}C_{\mathrm{met}}}+1}$$

where $D_{s}$ is the diffusivity of metal ions (Cu^+^) through the product layer, and $C_{\mathrm{met}}$ is the concentration of Cu^+^ on the Cu_2_O/CuO interface, which was considered to remain unchanged during the reaction. The initial condition for Equation (10) is $r_{2}=r_{2c}$. The grain size, $r_{1}$, is calculated with the following equation:

(11) $${r_{1}}^{3}={r_{2}}^{3}+Z\left({r_{0}}^{3}-{r_{2}}^{3}\right)$$

where $Z=2V_{\mathrm{CuO}}^{M}/V_{{\mathrm{Cu}}_{2}O}^{M}$ is the stoichiometric molar volume ratio of the solid product to the solid reactant, and $V_{\mathrm{CuO}}^{M}$ is the molar volume of CuO (12.52 cm^3^/mol).

The local conversion at each position inside the particle is calculated as

(12) $$\alpha =\left(1-\delta \right)\left(1-\frac{{r_{2}}^{3}}{{r_{0}}^{3}}\right)$$

The local porosity inside the particle during the reaction is calculated as a function of the initial porosity and the local conversion:

(13) $$\varepsilon =1-\left(1-\varepsilon_{0}\right)\cdot [1+\left(Z-1\right)\cdot \alpha ]$$

where ε is the local porosity, $\varepsilon_{0}$ is the initial porosity, and α is the local conversion.

### 2.3. Model at Particle Scale

Considering the external gas diffusion, internal gas diffusion, and chemical reaction, the O_2_ concentration profile inside the particle can be obtained by making a mass balance for the particle:

(14) $$\frac{1}{R^{2}}\frac{\partial }{\partial R}\left(R^{2}D_{e}\frac{\partial C}{\partial R}\right)=\frac{\left(1-\varepsilon_{0}\right)}{2V_{{\mathrm{Cu}}_{2}O}^{M}}\frac{\partial \alpha }{\partial t}$$

where R is the particle radius. The boundary conditions are as follows:

(15) $${\frac{\partial C}{\partial R}|}_{R=0}=0$$

(16) $${-D_{e}\frac{\partial C}{\partial R}|}_{R=R_{0}}=k_{g}\left(C_{s}-C_{0}\right)$$

where $R_{0}$ is the initial particle radius, and $C_{s}$ is the concentration of O_2_ on the particle external surface. The external mass transfer coefficient, $k_{g}$, is expressed as a function of Sherwood number:

(17) Sh=2kgR0DO2=2+0.664Re1/2Sc1/3

where Sh is the Sherwood number, Re is the Reynolds number, and Sc is the Schmidt number. $D_{O_{2}}$ is the gas molecular diffusivity and is given using the equation developed by Fuller et al. [40]:

(18) $$D_{O_{2}}=\frac{10^{-3}T^{1.75}{\left(\sum_{i=1}^{N}\frac{1}{M_{i}}\right)}^{0.5}}{P{\left[\sum_{i=1}^{N}{\left(v_{i}\right)}^{1/3}\right]}^{2}}$$

where M is the molar mass of the molecule, P is the pressure, and v is the diffusion volume for the molecule.

The effective gas diffusivity $D_{e}$ inside the porous particle is expressed as

(19) $$D_{e}=D_{1}\cdot \varepsilon^{2}$$

The gas diffusivity, $D_{1}$, is calculated as a combination of the gas molecular diffusivity and Knudsen diffusivity:

(20) $$D_{1}={\left({D_{O_{2}}}^{-1}+{D_{K}}^{-1}\right)}^{-1}$$

The Knudsen diffusivity, $D_{K}$, describes the collision of the gas molecules with the pore wall and is calculated as [41]

(21) $$D_{K}=\frac{4}{3}{\left(\frac{8R_{g}T}{\pi \cdot {\mathrm{MW}}_{O_{2}}}\right)}^{1/2}{\left[\frac{128}{9}\cdot \frac{3\left(1-\varepsilon_{0}\right)}{4\pi {r_{0}}^{3}}{r_{0}}^{2}\left(1+\frac{\pi }{8}\right)\right]}^{-1}$$

where $R_{g}$ is the universal gas constant. The overall particle conversion, $\bar{\alpha }$, can be obtained by integrating all local conversions:

(22) $$\bar{\alpha }=\frac{3}{{R_{0}}^{3}}\int_{0}^{R_{0}}R^{2}\alpha \cdot dR$$

## 3. Results

### 3.1. Effects of O_2_ Partial Pressure

**Figure 3:**
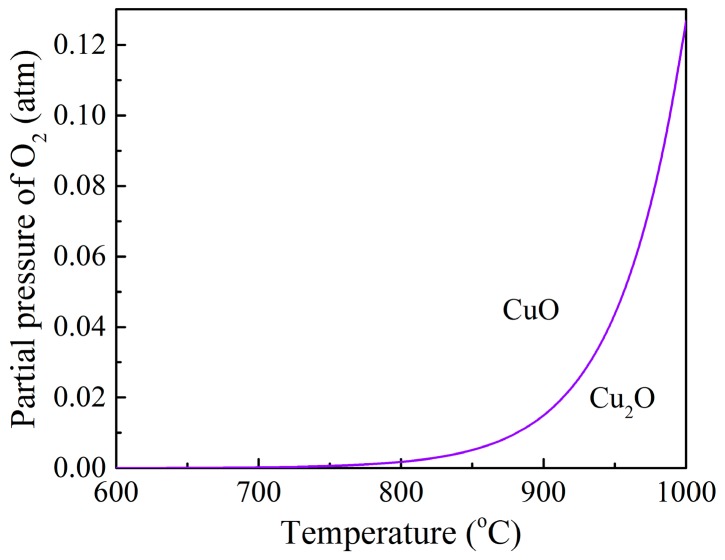
Equilibrium partial pressure of oxygen over the CuO/Cu_2_O redox pair.

The equilibrium curve of oxygen partial pressure in the oxidation reaction can be obtained using Equation (6), as shown in Figure 3. In high-O_2_ environments (such as the air reactor of a CLOU system), the Cu_2_O tends to be oxidized by O_2_, generating CuO. The driving force, which is defined as the difference between the actual partial pressure of oxygen and equilibrium partial pressure of oxygen [16], will affect the oxidation reaction rate of the oxygen carrier particle with O_2_. When the oxygen particle is in an atmosphere of air, the driving force will decrease with an increase in the temperature.

**Figure 4:**
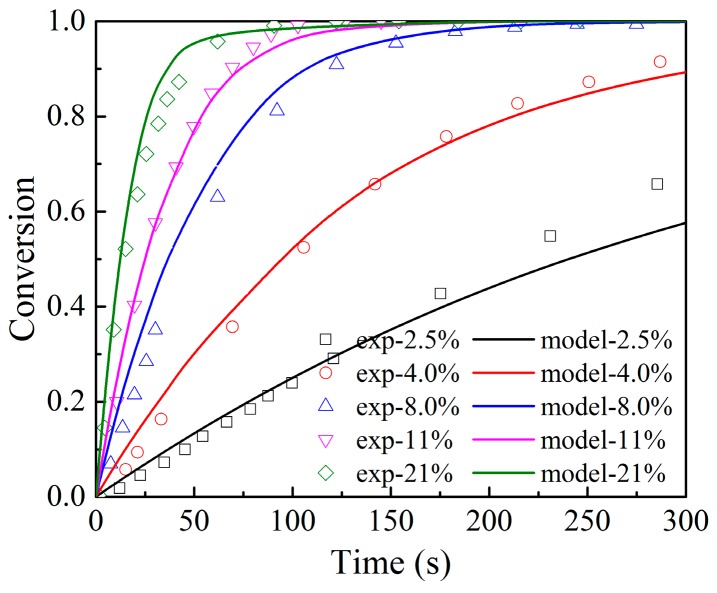
Effects of O_2_ volume percentage (vol%) on conversion at 900 °C. ([O_2_] = 2.5~21 vol%, N_2_ as equilibrium gas. Dots: experimental data [42]; lines: model results.)

The experimental data of the TGA in the study of Adánez-Rubio et al. [42] were used to validate the developed model in this study and analyze the effects of O_2_ partial pressure on the oxidation kinetics of Cu_2_O. In their experiment, the particles, prepared through calcination of 24 h at 1100 °C, had an average particle size of 200 μm and a porosity of 0.161, and the oxidation experiments were conducted at oxygen concentration values from 2.5 to 21 vol.% at 900 °C. The experimental data and modeling results were compared, as shown in Figure 4. It can be seen that the modeling results agree well with the experimental data and the developed multiscale model can predict the transition of the reaction kinetics from the initial fast stage to the second slower stage successfully. It is clear that the supplied oxygen partial pressure affects the particle conversion significantly. The oxidation reaction rate is fast when the oxygen partial pressure is much higher than the equilibrium oxygen partial pressure but decreases quickly with the decrease in the driving force. Whitty et al. [43] also observed this phenomenon.

**Figure 5:**
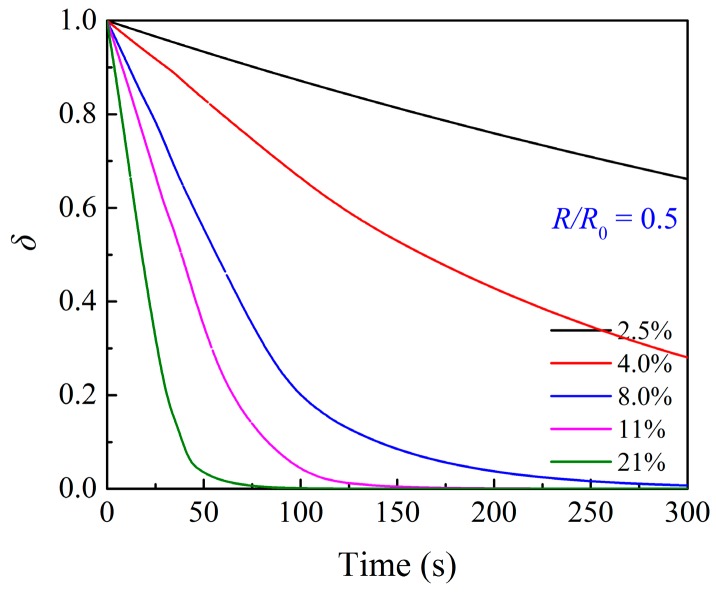
Profile of ratio of the unoccupied area on grain surface at *R*/*R*_0_ = 0.5 during the oxidation of the particle in Figure 4.

The profile of the ratio of the unoccupied area on the grain surface was plotted, as shown in Figure 5. It indicates that the transition of the conversion curve in Figure 4 happens when the product islands cover the grain surface. As observed in Figure 4, the duration time of the fast reaction stage will increase with the decrease in the supplied oxygen partial pressure. The reason is that the change rate of the ratio of the unoccupied area on the grain surface decreases with the decrease in the supplied oxygen partial pressure, as described in Equation (9). In the case of 2.5–4.0 vol.% O_2_, no transition of the conversion curve happens until 300 s; this is because there is still a part of unoccupied Cu_2_O surface which can react with O_2_ directly. In the case of 8.0–21 vol.% O_2_, the transition of the conversion curve occurs before 300 s when most part of the grain surface is covered by the product islands.

### 3.2. Effects of Temperature

The experimental data of TGA obtained from the research of Clayton et al. [16] were used to validate the developed model and investigate the effects of temperature on the oxidation reaction behavior. In their study, two different Cu-based carriers, named as 50_TiO_2_ material and 45_ZrO_2_ material, were tested under different temperatures. The 50_TiO_2_ material, supported by TiO_2_, was prepared using the mechanical mixing method, and CuO loading capacity was 50 wt.%. The 45_ZrO_2_ material, supported by ZrO_2_/MgO, was prepared using the freeze granulation method, and CuO loading capacity was 45 wt.%. To minimize mass transfer effects, small particle size (~40 μm) and a shallow layer of particles were used.

**Figure 6:**
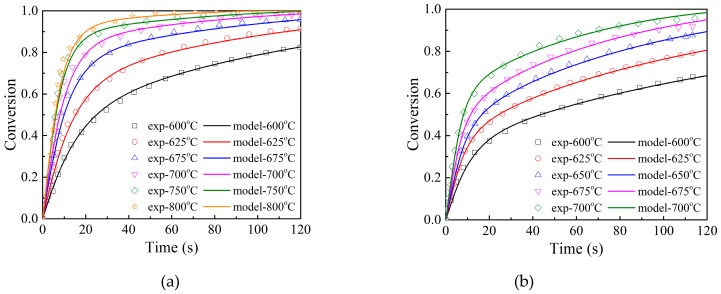
Effects of temperature on conversion of (**a**) 50_TiO_2_ material and (**b**) 45_ZrO_2_ material. ([O_2_] = 21 vol%, [N_2_] = 79 vol%. Dots: experimental data [16]; lines: calculated results.)

The calculated results using the multiscale model were compared with the TGA experimental results of Clayton et al. [16], as shown in Figure 6. Figure 6a shows conversion curves of a 50_TiO_2_ material particle under different temperatures (600–800 °C). Figure 6b shows conversion curves of a 45_ZrO_2_ material particle under different temperatures (600–700 °C). It shows that the conversion curves obtained from the model calculation agree well with the experimental data. The reaction rate in the initial fast stage is higher when the temperature is higher, although the increased amplitude gradually decreases with the increase in temperature. When the reaction time reaches 120 s, the final conversion increases with the increasing temperature. Comparing the reaction rate in the second lower reaction stage in Figure 6a,b, the reaction rate of a 50_TiO_2_ material particle is slower than that of the 45_ZrO_2_ material particle.

**Figure 7:**
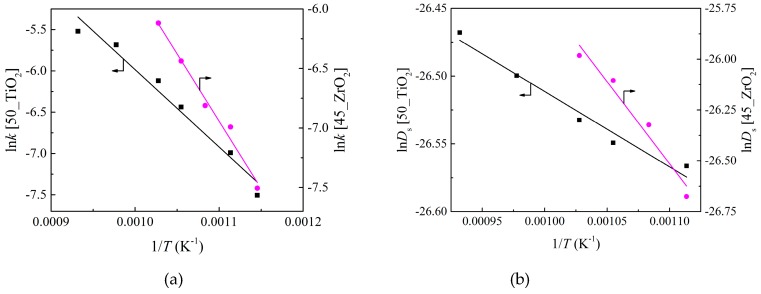

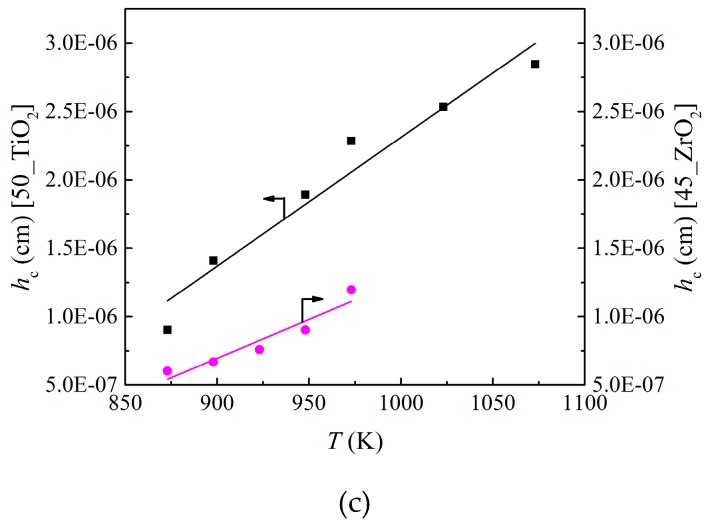
The relation curves of (**a**) ln*k* versus 1/*T*, (**b**) ln*D*_s_ versus 1/*T*, and (**c**) *h*_c_ versus *T*.

In the model calculation, there are three adjustable parameters included in the multiscale model: chemical reaction rate constant, *k*; the diffusivity of metal ions through the product layer, $D_{s}$; critical reactant layer thickness, *h*_c_. These parameters can be written as a function of temperature:

(23) $$k=k_{0}\exp\left(-\frac{E_{k}}{R_{g}T}\right)$$

(24) $$D_{s}=D_{0}\exp\left(-\frac{E_{D}}{R_{g}T}\right)$$

(25) $$h_{c}=aT+b$$

where $k_{0}$, $E_{k}$, $D_{0}$, $E_{D}$, a and b are kinetic parameters, and T is the temperature. The relation curves of these parameters obtained in the calculation of Figure 6 versus temperature were plotted, as shown in Figure 7. It can be seen from Equations (23)–(25) that the curves of ln*k* versus 1/*T*, ln*D*_s_ versus 1/*T*, and *h*_c_ versus *T* are linear, and the values of kinetic parameters can be obtained from Figure 7. The kinetic parameters were calculated and shown in Table 1.

**Figure 8:**
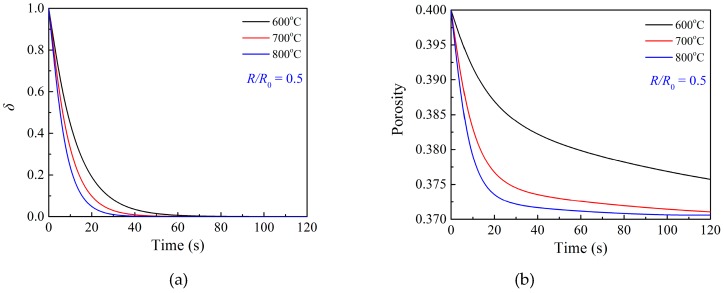
Profiles of (**a**) ratio of unoccupied area on grain surface at *R*/*R*_0_ = 0.5 and (**b**) local porosity at *R*/*R*_0_ = 0.5 during the 50_TiO_2__MM material oxidation reaction in Figure 6a.

Figure 8 presents the modeling output of the profiles of the ratio of the unoccupied area on grain surface and local porosity during the 50_TiO_2__MM material oxidation reaction in Figure 6a. It shows an increase of the temperature will result in a faster reduction of the ratio of the unoccupied area on grain surface because the critical solid reactant layer thickness increases with the increasing temperature [37]. Figure 8b shows the local porosity profile under different temperatures. The solid product has a larger molar volume than the solid reactant, giving rise to a progressive decrease of porosity throughout the sorbent particle. It is clear that the porosity significantly decreases in the initial fast stage, followed by a slight change in the second stage, which is attributed to the fact that product islands finally cover the grain surface, as shown in Figure 8a, and hence the product layer diffusion fully controls the reaction process.

### 3.3. Effects of Particle Structure

The particle structure has a significant influence on the oxidation reaction performance of an oxygen carrier with O_2_, including particle porosity, particle size, and grain size. The effects of these factors on oxidation performance were investigated using the model developed in this study.

**Figure 9:**
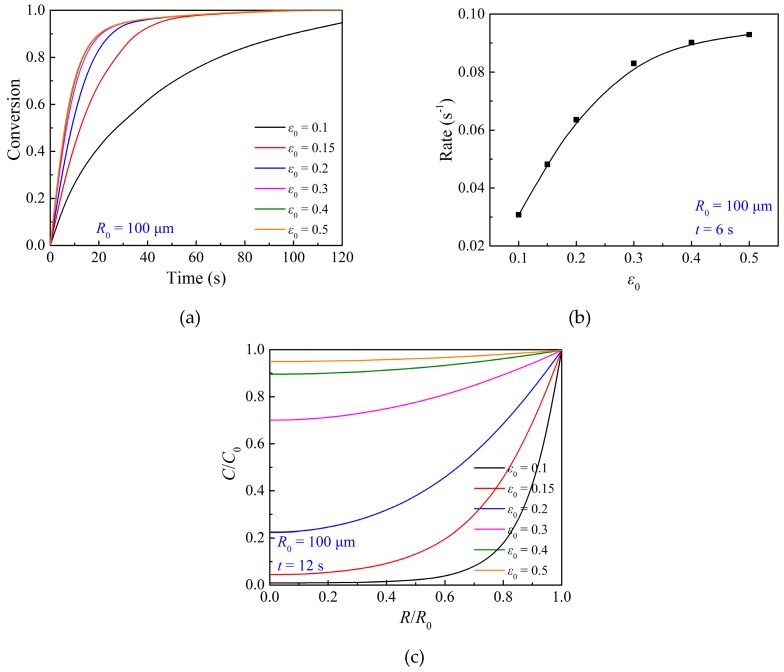
Effects of particle porosity on (**a**) the overall conversion of the particle, (**b**) reaction rate of the particle, and (**c**) oxygen concentration at *R*/*R*_0_ = 0.5 during the oxidation reaction at 800 °C.

The particle porosity plays an important role in the gas-solid reaction, and it is a key parameter considered in the preparation of an oxygen carrier particle. Particles with high porosity allow the reactant gas to achieve active sites easily, leading to a high reaction rate and overall conversion in specific residence time. Figure 9 presents the modeling results of the effects of porosity on the oxidation reaction. It can be seen that when the initial particle porosity is larger, the gas concentration inside the particle is higher, and the overall conversion of the corresponding particle is higher. However, as shown in Figure 9b, there is no further change in the reaction rate of the particle when the initial particle porosity is larger than 0.4, where the internal gas diffusion resistance is small enough inside the particle, as shown in Figure 9c. Moreover, the duration time of the initial fast reaction stage is influenced by the initial particle porosity, which is attributed to the effects of the particle porosity on the distribution of oxygen concentration inside the particle, as described in Equation (9).

**Figure 10:**
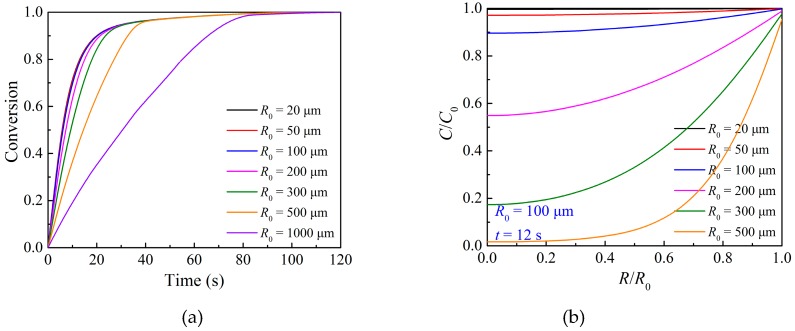
Effects of particle size on (**a**) the overall conversion of the particle and (**b**) oxygen concentration at *R*/*R*_0_ = 0.5 during the oxidation reaction at 800 °C.

The particle size usually affects the external and internal transfer of O_2_ significantly [8,44]. Figure 10 shows the modeling results of the effects of the particle size on the oxidation reaction. As can be seen, the decrease in the initial particle size will increase the reaction rate in the initial fast stage and the overall conversion of the particle. The corresponding oxygen concentration is higher with the decrease in the initial particle size. The radial distribution of oxygen concentration shows uniformity when the initial particle size is smaller than 100 μm, while there is a vast difference between the oxygen concentration at the particle center and that at the particle external surface when the initial particle size is larger than 300 μm. When the particle radius is smaller than 100 μm, a change in the particle size has no significant effect on the reaction performance, and the same conclusion was also reported in the research of García-Labiano [17]. This is because the internal gas diffusion resistance is small enough, as shown in Figure 10b, and there is a uniform reaction inside the particle, as also proposed by other researchers [45,46]. 

**Figure 11:**
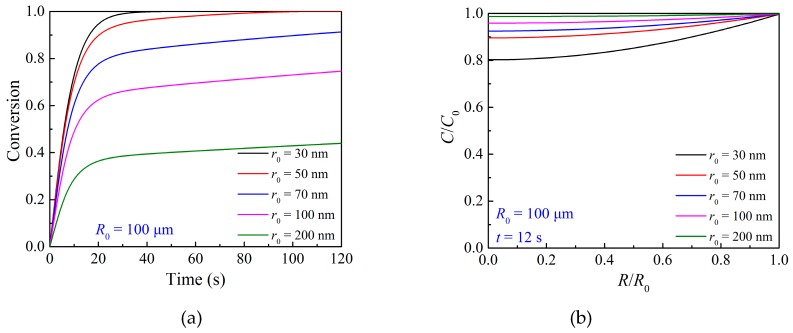
Effects of grain size on (**a**) the overall conversion of the particle and (**b**) oxygen concentration at *R*/*R*_0_ = 0.5 during the oxidation reaction at 800 °C.

The grain size determines the reaction interface and affects the oxidation process significantly. Figure 11a illustrates the overall conversion variation with the change of grain size as oxidation reaction proceeds. As smaller grains could provide more reaction interface areas, a smaller grain size results in a higher overall conversion in the same reaction time period. As observed, the particle with 30 nm grains could achieve the overall conversion up to 1.0 at 40 s, larger than two times of that of the particle with 200 nm grains. Therefore, the small grain size is desirable for high reaction performance of the oxygen carrier. It should be noted that the smaller grain size will lead to a small pore size, which will increase the gas internal diffusion resistance, as shown in Figure 11b.

## 4. Discussion

### 4.1. External Mass Transfer

The external mass transfer was considered in the particle scale model, and it might have an influence on the oxygen concentration in the external surface of the particle. As described in Equation (17), during the three particle structure parameters, only particle size affects the external mass transfer coefficient, $k_{g}$. Correspondingly, it is shown in Figure 10b that the particle size has an effect on the oxygen concentration in the particle external surface, while Figure 9c and Figure 11b show that the particle porosity and grain size have no effects on the oxygen concentration on the particle external surface. As observed in Figure 10b, even in the worst case simulated for the Cu_2_O oxidation reaction, where the particle size is up to 1 mm, the oxygen concentration on the particle external surface only decreased by 5% with respect to the ambient gas concentration.

**Figure 12:**
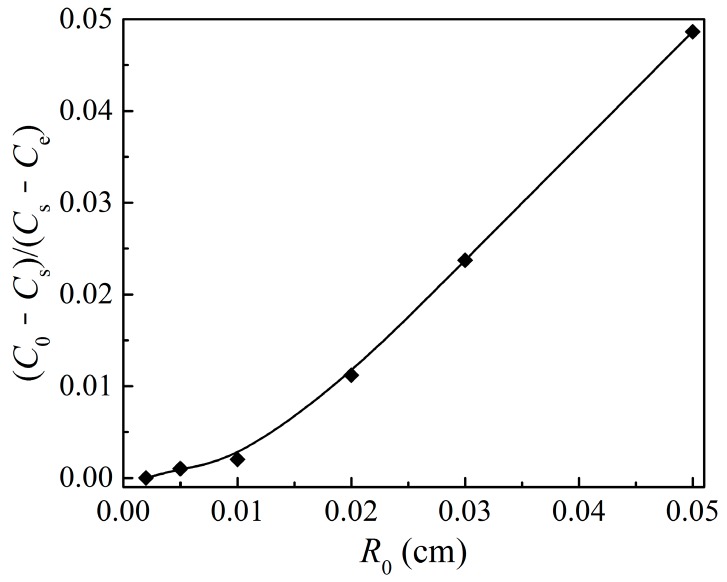
The importance of external mass transfer resistance described by $\left(C_{0}-C_{s}\right)/\left(C_{s}-C_{e}\right)$ under different particle sizes at 800 °C.

To analyze the effects of external mass transfer on the oxidation reaction behavior, the mass transfer rate of oxygen was analyzed. The mass transfer rate of oxygen to the particle is expressed as $4\pi {R_{0}}^{2}k_{g}\left(C_{0}-C_{s}\right)$. The reaction rate of the particle can be written as $4\pi {R_{0}}^{2}k_{\mathrm{int}}\left(C_{s}-C_{e}\right)$, where $k_{\mathrm{int}}$ is a comprehensive reaction rate constant describing the physical/chemical step inside the particle, including the internal gas diffusion, surface chemical reaction, and product layer diffusion. There should be $k_{g}\left(C_{0}-C_{s}\right)=k_{\mathrm{int}}\left(C_{s}-C_{e}\right)$. The magnitude of external mass transfer resistance can be described as $\left(1/k_{g}\right)/\left(1/k_{\mathrm{int}}\right)$, which is equal to $\left(C_{0}-C_{s}\right)/\left(C_{s}-C_{e}\right)$. Therefore, the value of $\left(C_{0}-C_{s}\right)/\left(C_{s}-C_{e}\right)$ was used to evaluate the importance of external mass transfer resistance during the oxidation reaction, as shown in Figure 12. It can be seen that even in the case of particle size of 1 mm, the importance of external mass transfer resistance is smaller than 0.05. Correspondingly, in the theoretical research of García-Labiano [17] and Sahir et al. [44], it was also predicted that the external mass transfer resistance hardly affected the reaction rate under the particle sizes normally used in a CLOU system, which was consistent with the calculation results in this work. In the experimental work of Chuang et al. [8], it was also proposed that the reaction of the particles (355–500 μm) was hardly controlled by external mass transfer. The external mass transfer resistance is small enough under the particle sizes investigated here, and it hardly affects the reaction performance. The oxidation behavior of the Cu_2_O particle is controlled by the physical/chemical step inside the particle.

### 4.2. Effectiveness Factor

The effectiveness factor, the ratio of the observed reaction rate to the intrinsic reaction rate, can be used to describe the relative importance of chemical reaction at grain scale versus internal gas diffusion inside the particle [47]. The chemical reaction step at grain scale includes the direct surface reaction on the unoccupied reactant surface and the indirect reaction on the solid product surface, where the reaction rate of the former step is very fast, and the reaction rate of the latter step is much slower.

The effectiveness factor, η, is expressed from a Thiele modulus, ϕ:

(26) $$\eta =\frac{3}{\phi^{2}}\left(\phi \coth\phi -1\right)$$

where

(27) $$\phi =\frac{\int_{0}^{R_{0}}4\pi R^{2}\varphi \cdot dR}{\frac{4}{3}\pi {R_{0}}^{3}}$$

(28) $$\varphi =R_{0}\sqrt{\frac{\gamma }{D_{e}}}$$

(29) $$\gamma =(1-\delta )\frac{3\frac{{r_{1}}^{2}}{{r_{0}}^{3}}(1-\varepsilon_{0})k}{r_{1}(\frac{r_{1}}{r_{2}}-1)\frac{k\left(C-C_{e}\right)}{D_{s}C_{\mathrm{si}}}+1}+\delta (1-\frac{{r_{2}}^{3}}{{r_{0}}^{3}})\frac{(1-\varepsilon_{0})k}{h_{c}}$$

**Figure 13:**
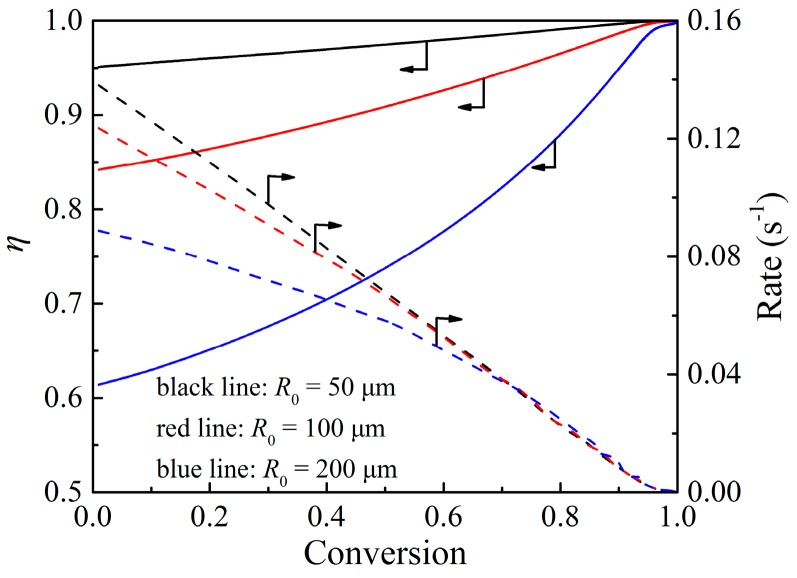
Calculated results of effectiveness factor and reaction rate against particle conversion under different particle sizes at 800 °C.

The value of the effectiveness factor changes during the reaction process. For the oxidation of the Cu_2_O, the stoichiometric molar volume ratio of the solid product to the solid reactant is Z=1.05, which is slightly larger than one. It means that there is just a slight decrease in the porosity inside the particle, as proved in Figure 8b. Therefore, the change of internal gas diffusion resistance during the reaction process is not obvious. The chemical reaction at grain scale is the main factor to affect the value of the effectiveness factor during the reaction process. Figure 13 shows the calculated results of the effectiveness factor and reaction rate against particle conversion under different particle sizes normally used in a CLOU system. As can be seen, under the particle radius of 50 μm, the effectiveness factor remains quite high throughout the entire reaction process, which means the whole oxidation reaction process is under the control of the chemical reaction at grain scale. The value of the effectiveness factor decreases with the increase in the particle size, which means the internal gas diffusion resistance is higher inside a particle with larger particle size, and the corresponding reaction rate decreases when the particle size increases. Under a certain particle size, as the reaction goes by, the value of the effectiveness factor increases, and the reaction rate decreases, which means chemical reaction resistance at grain scale increases. During the oxidation reaction process, the product islands grow quickly to cover the grain surface, leading to the reaction process changing from fast reaction stage to the second slower stage. Therefore, the increase in the chemical reaction resistance at grain scale results in the increase of the effectiveness factor.

## 5. Conclusions

A multiscale model was established to describe the Cu-based oxygen carrier oxidation reaction with oxygen in this paper, including the surface scale, grain scale, and particle scale. The effects of oxygen partial pressure, temperature, and particle structure on the oxidation kinetics were studied using the developed model. The modeling results indicate that the transition of the conversion curve occurs when product islands cover most part of the grain surface. The oxygen partial pressure and particle structure have an obvious influence on the duration time of the fast reaction stage. An increase of the particle porosity, a decrease of the particle size, or a decrease of grain size will lead to better oxidation reaction behavior. However, no significant effect on the reaction was found when the initial particle porosity is larger than 0.4, or the initial particle radius is smaller than 100 μm, which is due to the negligible gas diffusion resistance. Furthermore, the importance of the external mass transfer and the effectiveness factor during the oxidation reaction process under the particle sizes normally used in a CLOU system were discussed to investigate the controlling step of the oxidation reaction of Cu-based oxygen carrier. It was concluded that the external mass transfer step hardly affects the reaction performance, and the oxidation behavior of the Cu_2_O particle is controlled by the physical/chemical step inside the particle. The value of the effectiveness factor increases as the reaction goes by, which means the increased chemical reaction resistance at grain scale results from the growing number of product islands on the grain surface. Moreover, the multiscale model developed in this paper is expected to be useful for the oxidation kinetic analysis of other oxygen carriers used in CLOU.

## Figures and Tables

**Table 1:** Values of the kinetic parameters for the oxidation of Cu-based oxygen carrier.

Parameter	*k*_0_ (cm/s)	*E*_k_ (kJ/mol)	*D*_0_ (cm^2^/s)	*E*_D_ (kJ/mol)	*a* (cm/K)	*b* (cm)
50_TiO_2_ material	29	78	5.3 × 10^−12^	4.6	9.4 × 10^−9^	−7.1 × 10^−6^
45_ZrO_2_ material	245	94	2.2 × 10^−8^	67	5.7 × 10^−9^	−4.4 × 10^−6^

## Nomenclature

*C*	concentration of O_2_ inside the particle, mol/cm^3^
*C*_0_	concentration of O_2_ in the ambient gas, mol/cm^3^
*C*_e_	equilibrium concentration of O_2_, mol/cm^3^
*C*_met_	concentration of metal ions on the Cu_2_O/CuO interface, mol/cm^3^
*C*_s_	concentration of O_2_ on the particle external surface, mol/cm^3^
*D*_1_	diffusivity of gas through the particle pores, cm^2^/s
*D*_e_	effective diffusivity of gas through the particle pores, cm^2^/s
*D*_K_	Knudsen diffusivity, cm^2^/s
$D_{O_{2}}$	O_2_ molecular diffusivity, cm^2^/s
*D*_s_	diffusivity of metal ions through the product layer, cm^2^/s
*E*_k_, *E*_D_	activation energy, kJ/mol
*h*_c_	critical solid reactant layer thickness, cm
*k*	chemical reaction rate constant, cm/s
*k*_g_	external mass transfer coefficient, cm/s
P	pressure, atm
*P*_e_	equilibrium partial pressure of O_2_, atm
*r*	grain radius, cm
*r*_0_	initial grain radius, cm
*r*_1_	grain radius of CuO/O_2_ interface, cm
*r*_1c_	critical grain radius of CuO/O_2_ interface, cm
*r*_2_	grain radius of Cu_2_O/CuO interface, cm
*r*_2c_	critical grain radius of Cu_2_O/CuO interface, cm
*R*	particle radius, cm
*R*_0_	initial particle radius, cm
*R*_g_	the universal gas constant, J/(mol·K)
*t*	reaction time, s
*T*	temperature, K
*v*	diffusion volume for molecule, cm^3^
$V_{\mathrm{CuO}}^{M}$	molar volume of CuO, cm^3^/mol
$V_{{\mathrm{Cu}}_{2}O}^{M}$	molar volume of Cu_2_O, cm^3^/mol
*Z*	stoichiometric molar volume ratio of the solid product to the solid reactant
*α*	local conversion
$\overset{-}{\alpha }$	overall conversion
*δ*	ratio of the unoccupied area on the grain surface
*ε*	local porosity
*ε*_0_	initial porosity
η	effectiveness factor
ϕ	Thiele modulus
